# Supplementary material for: Unravelling the Link between Psychological Distress and Liver Disease: Insights from an Anxiety-like Rat Model and Metabolomics Analysis
Source: Int J Mol Sci. 2023 Aug 29;24(17):13356. doi: 10.3390/ijms241713356 (PMC10487641; doi:10.3390/ijms241713356)
Supplement: Supplementary file 1 [file ijms-24-13356-s001.zip › ijms-2524605-supplementary.pdf]

Supplementary Materials for

Unravelling the link between psychological distress and liver disease: Insights from an anxiety-like rat model and metabolomics analysis

Supplementary Figure Legends

Supplementary Figure S1. Overview of instrument stability. (A) PCA scatter plot of the QCs in negative ion mode; (B) PCA scatter plot of the QCs in positive ion mode.

Supplementary Figure S2. Overview of plasma metabolomics in study samples. (A) PCA scatter plot of metabolic profiles in positive ion mode. (B) PCA scatter plot of metabolic profiles in negative ion mode.

Supplementary Figure S3. (A) Permutation tests corresponding to OPLS-DA model in positive ion mode; (B) Permutation tests corresponding to OPLS-DA model in negative ion mode.

Supplementary Figures

Supplementary Figure S1

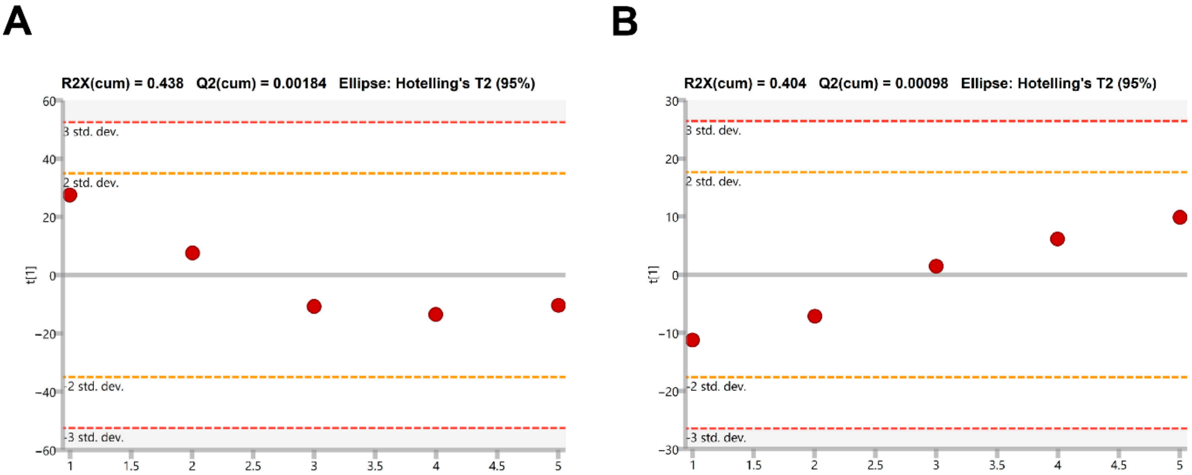

Supplementary Figure S2

A **B**

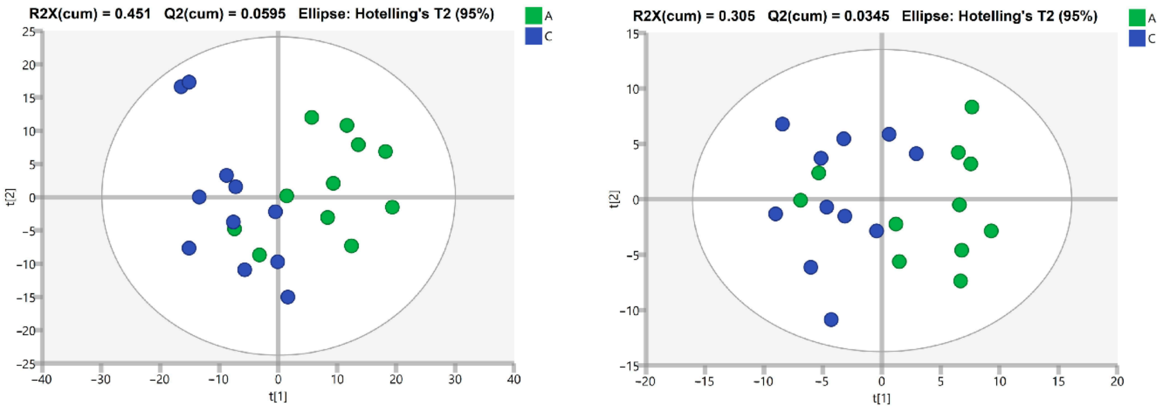

Supplementary Figure S3

A

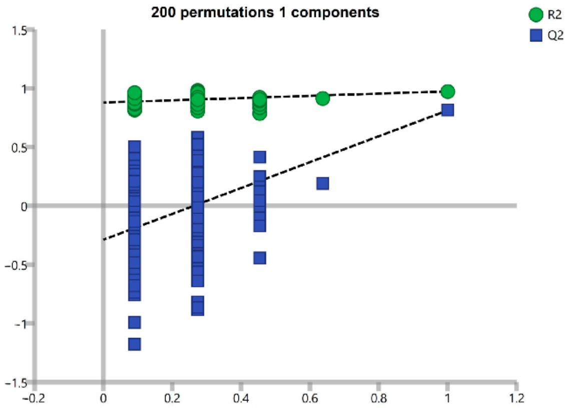

B

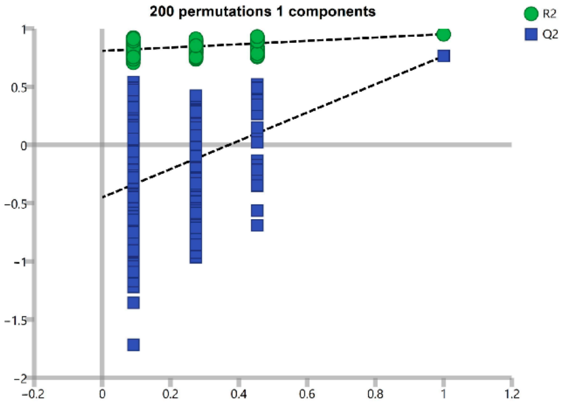

Supplementary Tables

Supplementary Table S1

Antibodies to list

Antibody	merchant	Article number	dilution rate
Phospho-EGF Receptor (Tyr1068) (D7A5)	Cell Signaling Technology	#3777	1:1000
EGFR	PTMBIO	PTM-5042	1:1000
Phospho-PI3K p85 alpha (Tyr607)	Affinity	AF3241	1:1000
PI3 Kinase p85 alpha	Abmart	T40115F	1:1000
Phospho-Akt (Thr308) (D25E6)	Cell Signaling Technology	#13038	1:1000
anti-AKT	Abmart	T55561F	1:1000
Phospho-IKB alpha (Ser32/Ser36)	Affinity	AF2002	1:1000
IKB alpha	Servicebio	GB13212-1	1:1000
Phospho-NF-κB p65 (Ser536)	Affinity	AF2006	1:1000
NF-κB p65	Servicebio	GB11997	1:1000
Phospho- NF kappaB p105/p50 (Ser337)	Affinity	AF3219	1:1000
NF kappaB p105/p50	Affinity	AF6217	1:1000
TNF-α (52B83)	Santa Cruz Biotechnology	Sc-52746	1:500
IL-1β (B122)	Santa Cruz Biotechnology	Sc-12742	1:500
IL-6 (D5W4V)	Cell Signaling Technology	#12912	1:1000
Anti-rabbit lgG, HRP-linked Antibody	Cell Signaling Technology	#7074	1:2000
Goat Anti-Mouse IgG (H+L) HRP	Servicebio	GB23301	1:2000

Supplementary Table S2

Primers used for qPCR testing

Gene symbol	direction	Gene sequence
EGFR	Forward	TCCCAAAGAAGCCAAGCCGAATG
	Reverse	TGCCTCTTCAATGTCATGCTCCAC
PI3KR1	Forward	GTGGTAGATGGCGAAGTCA
	Reverse	CAGGGAGGTGTGTTGGTAA
AKT1	Forward	ATTGAGCGCACCTTCCA
	Reverse	TTTGAGTCCATCAGCCACA
NFKBIA	Forward	TGAAGGACGAGGATTACGA
	Reverse	CCAAGTGCAGGAACGAG
NFκB1	Forward	CCCCTGAGAAAGAAACACA
	Reverse	CCTGAAACCCCACATCC
RELA	Forward	ATCCAAAGCAACGCTCCTA
	Reverse	CAATGTCCCCTCCTGGTC
GAPDH	Forward	GACATGCCGCCTGGAGAAAC
	Reverse	AGCCCAGGATGCCCTTTAGT

Supplementary Table S3

The differential metabolites between Anxiety and Control

Compound	Mass	RT (min)	P value	VIP	Log2FC	HMDB	Mode
1,3-Dimethyluracil	140.0593	1.35	2.00E-03	1.57	-0.16	HMDB0002144	Pos
Bis(2-hydroxypropyl) amine	133.1104	0.96	1.00E-03	1.68	-0.52	HMDB0251354	Pos
(R)-1-O-[b-D-Glucopyranosyl-(1->6)-b-D-glucopyranoside]-1,3-octanediol	470.2368	6.28	0.00E+00	1.92	0.30	HMDB0032799	Pos
n-Butyl-2-cyanoacrylate	153.0782	1.41	1.00E-03	1.80	-0.43	HMDB0249472	Pos
Choline	104.1075	1.45	1.00E-03	1.62	-0.53	HMDB0000097	Pos
2-Hydroxy-dAMP	347.063	1.40	3.00E-03	1.53	-0.30	HMDB0059594	Pos
Pirimicarb	238.1415	3.80	3.00E-02	1.10	-0.40	HMDB0256598	Pos
PS(P-20:0/22:6(4Z,7Z,10Z,13Z,16Z,19Z))	847.5735	16.80	1.10E-02	1.28	0.53	HMDB0256890	Pos
Glutamyl-asparagine	261.0957	0.97	7.00E-03	1.48	-0.45	HMDB0028814	Pos
2-Acetyl-1,5,6,7-tetrahydro-6-hydroxy-7-(hydroxymethyl)-4H-azepine-4-one	199.0838	1.41	0.00E+00	1.92	-0.53	HMDB0035177	Pos
N-(1-Deoxy-1-fructosyl)serine	267.0954	1.44	1.90E-02	1.30	-0.41	HMDB0037842	Pos
Osmaronin	259.103	3.32	0.00E+00	1.92	-0.79	HMDB0032769	Pos
2-Methylpropyl formate	102.0682	0.96	4.10E-02	1.24	-0.30	HMDB0031247	Pos
3-Hydroxy-2-pentanone	102.0677	1.46	2.00E-03	1.58	-0.59	HMDB0031516	Pos
Ganoderic acid H	572.2979	12.82	1.00E-03	1.73	-1.43	HMDB0035987	Pos
Glucosyl passiflorate	696.4122	5.85	3.00E-02	1.17	-0.22	HMDB0038141	Pos
p-Anisaldehyde	136.0516	1.41	0.00E+00	1.88	-0.44	HMDB0029686	Pos
Sulfolane	120.0255	9.44	4.50E-02	1.05	-0.38	HMDB0258594	Pos
Methionine	149.0508	1.35	0.00E+00	1.88	-0.53	HMDB0002005	Pos
Asp Val	232.1049	1.39	4.00E-02	1.23	-0.44	HMDB0028766	Pos
Threoninyl-Valine	218.1276	8.02	2.00E-03	1.34	-0.23	HMDB0304791	Pos
5-Hexyltetrahydro-2-oxo-3-furancarboxylic acid	214.1198	7.49	9.00E-03	1.37	0.27	HMDB0030984	Pos
Naringenin 7-O-glucoside	434.1212	3.87	5.00E-03	1.53	-0.31	HMDB0304708	Pos
Gabapentin	171.1257	5.37	3.10E-02	1.04	-0.41	HMDB0005015	Pos
Alizapride	315.1671	3.53	3.30E-02	1.19	-0.44	HMDB0015494	Pos
Spermidine	145.1573	0.80	3.50E-02	1.19	0.41	HMDB0001257	Pos

Staurosporine	466.2019	14.37	1.60E-02	1.34	-0.32	HMDB0247152	Pos
Acetone oxime	73.0531	1.35	1.00E-03	1.79	-0.37	HMDB0247913	Pos
2-Methylcitrate	206.0428	0.83	4.60E-02	1.33	-0.21	HMDB0000379	Pos
Pivaloylcarnitine	246.1697	4.49	1.70E-02	1.30	-0.39	HMDB0041993	Pos
Fludioxonil	248.0396	1.33	5.00E-03	1.42	-0.34	HMDB0252328	Pos
Cyclocreatine	143.0692	1.38	2.00E-03	1.66	-0.31	HMDB0250642	Pos
N-palmitoyl glutamine	384.2972	12.93	4.00E-03	1.68	-0.43	HMDB0241924	Pos
Benserazide	257.1021	11.59	4.80E-02	1.06	0.14	HMDB0248960	Pos
Indoramin	347.2019	3.80	4.70E-02	1.24	-0.31	HMDB0253479	Pos
Decanoylcholine	258.2425	9.83	0.00E+00	1.86	-0.22	HMDB0013228	Pos
Acrimarine H	513.1812	0.97	1.90E-02	1.42	0.37	HMDB0038599	Pos
Heptyl acetate	158.13	8.92	1.00E-03	1.25	-0.14	HMDB0031480	Pos
1-Pyrroline-5-carboxylic acid	113.0478	1.35	1.00E-03	1.67	-0.22	HMDB0001301	Pos
Dihydrojasmonic Acid, Methyl Ester	226.1562	10.72	3.00E-03	1.56	-0.18	HMDB0031740	Pos
6,8-Dihydroxypurine	152.0343	1.36	0.00E+00	2.00	-0.44	HMDB0011473	Neg
D-Pantothenic acid	219.1111	3.31	8.00E-03	1.59	-0.39	HMDB0000210	Neg
Acetic acid	60.0207	0.94	3.10E-02	1.50	-0.50	HMDB0000042	Neg
Mammeigin	404.1642	14.37	1.40E-02	1.19	0.69	HMDB0030785	Neg
Scarlet Red	380.1645	14.54	7.00E-03	1.33	0.94	HMDB0258560	Neg
LysoPE(18:1(11Z)/0:0)	479.3003	12.63	4.10E-02	1.26	0.53	HMDB0011505	Neg
LysoPE(24:6(6Z,9Z,12Z, 15Z,18Z,21Z)/0:0)	553.3154	11.70	3.50E-02	1.19	0.28	HMDB0011529	Neg
Furathiocarb	382.1587	14.16	5.00E-03	1.34	0.66	HMDB0252533	Neg
Dephospho-CoA	687.1473	3.20	1.90E-02	1.24	0.58	HMDB0001373	Neg
Erythronic acid	136.0373	1.05	2.60E-02	1.17	-0.50	HMDB0000613	Neg
Nicotinamide riboside	255.0986	2.84	4.90E-02	1.40	0.61	HMDB0000855	Neg
N-Acetyl-4-O-acetylneuraminic acid	351.1162	1.40	0.00E+00	1.82	-0.51	HMDB0000796	Neg
Meta-Tyrosine	181.074	1.41	1.00E-03	1.85	-0.34	HMDB0059720	Neg
1-O-Galloyl-beta-D-glucose	332.0717	6.65	3.50E-02	1.17	-0.64	HMDB0301708	Neg
Riboflavin	376.1372	4.59	2.10E-02	1.47	-0.28	HMDB0000244	Neg
Flavine mononucleotide (FMN)	456.1041	4.20	2.00E-03	1.43	-0.19	HMDB0001520	Neg
PS(18:0/0:0)	525.3056	14.39	2.00E-03	1.61	-0.74	HMDB0240606	Neg
Adenosine 2'-phosphate	347.064	1.34	7.00E-03	1.58	2.09	HMDB0011617	Neg
Fagomine	147.0896	3.31	1.50E-02	1.66	-0.37	HMDB0033453	Neg
Linoleoyl glycine	337.2613	13.29	9.00E-03	1.71	-0.33	HMDB0241917	Neg
L-Homoserine lactone	101.0479	0.92	5.00E-02	1.33	-0.30	HMDB0255213	Neg

Supplementary Statistics Table S4

The differential pathways between Anxiety and Control

Metabolic pathway	Differential metabolites	P value
Riboflavin metabolism	Riboflavin; FMN	6.71E-04
Pantothenate and CoA biosynthesis	Dephospho-CoA；Pantothenate	1.74E-02
Arginine and proline metabolism	Spermidine; (S)-1-Pyrroline-5-carboxylate	6.32E-02
Nicotinate and nicotinamide metabolism	Nicotinamide-beta-riboside	1.53E-01
beta-Alanine metabolism	Spermidine	2.08E-01
Pyruvate metabolism	Acetate	2.17E-01
Glycolysis / Gluconeogenesis	Acetate	2.51E-01
Alanine, aspartate and glutamate metabolism	(S)-1-Pyrroline-5-carboxylate	2.68E-01
Glutathione metabolism	Spermidine	2.68E-01
Glyoxylate and dicarboxylate metabolism	Acetate	3.00E-01
Glycine, serine and threonine metabolism	Choline	3.08E-01
Glycerophospholipid metabolism	Choline	3.31E-01

Supplementary Table S5

Statistical analysis of changes in anxiety index after stress

Group	Number of examples	*M(P25~P75)*	*z*	*p*
Control	8	80.872(74.582,82.494)	-2.205	0.027
Anxiety	8	86.474(80.982,89.531)		

The normality test results of group C and group A were 0.048 and 0.237, respectively, which did not meet the normality, and the homogeneity test result was 0.838, which satisfied the homogeneity of variance, and the difference was statistically significant by using two independent samples of nonparametric Mann-Whitney U, $p<0.05$.

Statistical analysis of the change in total distance of action in post-stress open field experiments

Group	Number of examples	$\bar{x}\pm s$	*t*	*p*
Control	8	7.605±2.585	1.129	0.278
Anxiety	8	6.243±2.225		

The normality test results of group C and group A were 0.723 and 0.407, respectively, satisfying normality, and the homogeneity of variance test result was 0.678, satisfying homogeneity of variance, and two independent samples were used t-test, $P<0.05$, which was statistically significant.

Statistical analysis of time variation in the central region of post-stress open field experiments

Group	Number of examples	$\bar{x}\pm s$	*t*	*p*
Control	8	13.029±7.857	2.454	0.028
Anxiety	8	5.023±4.841		

The normality test results of group C and group A were 0.275 and 0.320, respectively, satisfying normality, and the homogeneity test result was 0.052, satisfying homogeneity of variance, and two independent samples were used t-test, $P<0.05$, which was statistically significant.

Statistical analysis of immobility time variation in forced swimming experiment after stress

Group	Number of examples	$\bar{x}\pm s$	*t*	*p*
Control	8	36.501±4.523	12.960	0.000
Anxiety	8	10.098±3.570		

The normality test results of group C and group A were 0.965 and 0.669, respectively, satisfying normality, and the homogeneity test result was 0.572, satisfying the homogeneity of variance, and the $p<0.05$ was used for the t-test of two independent samples, which was statistically significant.

Statistical analysis of sugar water preference rate change in

sugar water preference experiment after stress

Group	Number of examples	*M(P25~P75)*	*z*	*p*
Control	8	96.776(87.858,99.722)	-3.363	0.001
Anxiety	8	42.222(26.608,52.228)		

The normality test results of group C and group A were 0.041 and 0.694, respectively, which did not meet the normality, and the homogeneity of variance test result was 0.108, which satisfied the homogeneity of variance, and two independent samples were used nonparametric Mann-Whitney U, $p<0.05$, which were statistically significant.

Statistical analysis of weight change during animal experiments

Group	Number of examples	$\bar{x}\pm s$	*t*	*p*
Control	8	91.787±7.250	-7.896	0.000
Anxiety	8	49.250±13.401		

The normality test results of group C and group A were 0.275 and 0.112, respectively, satisfying normality, and the homogeneity test result was 0.153, satisfying the homogeneity of variance, and the test of two independent samples was used, $p<0.05$, which was statistically significant.

Statistical analysis of liver coefficient changes in animals

Group	Number of examples	$\bar{x}\pm s$	*t*	*p*
Control	8	2.912±0.596	9.695	0.000
Anxiety	8	3.445±0.145		

The normality test results of group C and group A were 0.266 and 0.729, respectively, satisfying normality, and the homogeneity test result was 0.068, satisfying the homogeneity of variance, and the $p<0.05$ was used for the t-test of two independent samples, which was statistically significant.

Statistical analysis of changes in ALP viability in animal serum

Group	Number of examples	*M(P25~P75)*	*z*	*p*
Control	8	17.934(15.279,20.127)	-3.151	0.002
Anxiety	8	22.022(20.460,25.141)		

The normality test results of group C and group A were 0.141 and 0.042, respectively, which did not meet the normality, and the homogeneity of variance test result was 0.441, which satisfied the homogeneity of variance, and two independent samples were used nonparametric Mann-Whitney U, $p<0.05$, and the difference was statistically significant.

Statistical analysis of changes in AST viability in animal serum

Group	Number of examples	*M(P25~P75)*	*z*	*p*
Control	8	23.522(18.621,29.463)	-3.361	0.001
Anxiety	8	61.888(42.697,84.061)		

The normality test results of group C and group A were 0.794 and 0.125, respectively, satisfying normality,

and the homogeneity test result was 0.005, which did not meet the homogeneity of variance, and two independent samples were used nonparametric Mann-Whitney U, $p<0.05$, which were statistically significant.

Statistical analysis of changes in ALT viability in animal serum

Group	Number of examples	*M(P25~P75)*	*z*	*p*
Control	8	25.278(21.061,32.654)	-3.361	0.001
Anxiety	8	47.218(42.735,65.748)		

The normality test results of group C and group A were 0.719 and 0.111, respectively, satisfying normality, and the homogeneity test result was 0.018, which did not meet the homogeneity of variance, and two independent samples were used nonparametric Mann-Whitney U, $p<0.05$, which were statistically significant.

Statistical analysis of changes in AST/ALT ratio in animal serum

Group	Number of examples	*M(P25~P75)*	*z*	*p*
Control	8	0.944(0.834,1.022)	-2.415	0.016
Anxiety	8	1.161(1.043,1.438)		

The normality test results of group C and group A were 0.712 and 0.577, respectively, satisfying normality, and the homogeneity test result was 0.023, which did not meet the homogeneity of variance, and two independent samples were used nonparametric Mann-Whitney U, $p<0.05$, which were statistically significant.

Statistical analysis of area of inflammatory infiltrates in HE staining

Group	Number of examples	*M(P25~P75)*	*z*	*p*
Control	8	9927.092(7215.988,16496.312)	-3.361	0.001
Anxiety	8	113934.060(79969.061,201185.626)		

The normality test results of group C and group A were 0.249 and 0.047, respectively, which did not meet the normality, and the homogeneity test result was 0.000, which did not meet the homogeneity of variance, and two independent samples were used nonparametric Mann-Whitney U, $p<0.05$, which were statistically significant.

Statistical analysis of changes in CRH content in animal serum

Group	Number of examples	$\bar{x}\pm s$	*t*	*p*
Control	6	13.706±1.005	2.237	0.049
Anxiety	6	15.690±1.925		

The normality test results of group C and group A were 0.132 and 0.125, respectively, satisfying normality, and the homogeneity of variance test result was 0.051, satisfying the homogeneity of variance, and the difference was statistically significant by using two independent samples t-test, $p<0.05$.

Statistical analysis of changes in ACTH content in animal serum

Group	Number of examples	$\bar{x}\pm s$	*t*	*p*

Control	6	18.182±0.570	-2.903	0.016
Anxiety	6	18.950±0.309		

The normality test results of group C and group A were 0.139 and 0.637, respectively, satisfying normality, and the homogeneity test result was 0.084, satisfying homogeneity of variance, and two independent samples were used t-test, $p<0.05$, which was statistically significant.

Statistical analysis of changes in CORT content in animal serum

Group	Number of examples	$\bar{x}\pm s$	t	p
Control	6	5.723±1.130	6.502	0.000
Anxiety	6	10.487±1.395		

The normality test results of group C and group A were 0.365 and 0.392, respectively, satisfying normality, and the homogeneity test result was 0.457, satisfying the homogeneity of variance, and the test of two independent samples was used, $p<0.05$, and the difference was statistically significant.

Statistical analysis of changes in CRH content in animal brains

Group	Number of examples	*M(P25~P75)*	z	p
Control	6	16.920(16.363,18.009)	-2.242	0.025
Anxiety	6	19.474(19.086,20.335)		

The normality test results of group C and group A were 0.021 and 0.403, respectively, which did not meet the normality, and the homogeneity of variance test result was 0.361, which satisfied the homogeneity of variance, and two independent samples were used nonparametric Mann-Whitney U, $p<0.05$, and the difference was statistically significant.

Statistical analysis of changes in ACTH content in animal brains

Group	Number of examples	$\bar{x}\pm s$	t	p
Control	6	20.052±1.036	4.123	0.002
Anxiety	6	22.541±1.055		

The normality test results of group C and group A were 0.777 and 0.512, respectively, satisfying normality, and the homogeneity test result was 0.767, satisfying homogeneity of variance, and two independent samples were used t-test, $p<0.05$, which was statistically significant.

Statistical analysis of changes in inflammatory factor protein content in animal brains

Group	Protein	Number of examples	$\bar{x}\pm s$/*M(P25~P75)*	*t/z*	p
Control	TNF	6	0.354±0.050	-5.272	0.000
Anxiety		6	0.605±0.106		
Control	IL-1β	6	0.465(0.456,0.540)	-2.882	0.004
Anxiety		6	0.931(0.703,1.155)		
Control	IL-6	6	0.623(0.540,0.681)	-2.882	0.004
Anxiety		6	0.807(0.687,1.000)		

The normality test results of TNF protein in group C and group A were 0.266 and 0.257, respectively, which

met the normality, and the homogeneity test result was 0.151, which satisfied the homogeneity of variance, and the difference was statistically significant by using two independent samples t-test, $p<0.05$. The normality test results of IL-1β protein in group C and group A were 0.022 and 0.726, respectively, which did not meet the normality, and the homogeneity test result was 0.039, which did not meet the homogeneity of variance, and two independent samples were used as nonparametric Mann-Whitney U, $p<0.05$, and the difference was statistically significant. The results of IL-6 protein normality test in group C and group A were 0.082 and 0.007, respectively, which did not meet the normality, and the homogeneity test result was 0.155, satisfying the homogeneity of variance, and two independent samples were used nonparametric Mann-Whitney U, $p<0.05$, and the difference was statistically significant.

Statistical analysis of changes in the content of inflammatory factors in the brain of animals

Group	Protein	Number of examples	$\bar{x}\pm s/M(P25\sim P75)$	*t/z*	*p*
Control	TNF	5	12.682(12.171,13.273)	-2.402	0.016
Anxiety		5	15.690(13.620,27.790)		
Control	IL-1β	5	1.361(1.303,1.644)	-1.984	0.047
Anxiety		5	1.736(1.486,3.363)		
Control	IL-6	5	16.472±2.074	-3.945	0.004
Anxiety		5	23.690±3.527		

The normality test results of TNF expression level in group C and group A were 0.118 and 0.636, respectively, which satisfied the normality, and the homogeneity test result was 0.030, which did not meet the homogeneity of variance, and two independent samples were used nonparametric Mann-Whitney U, $p<0.05$, which were statistically significant. The results of IL-1β protein normality test in group C and group A were 0.118 and 0.008, respectively, which did not meet the normality, and the homogeneity test result was 0.050, which did not meet the homogeneity of variance, and two independent samples were used nonparametric Mann-Whitney U, $p<0.05$, and the difference was statistically significant. The results of IL-6 protein normality test in group C and group A were 0.236 and 0.224, respectively, satisfying normality, and the homogeneity test result was 0.336, satisfying homogeneity of variance, and two independent samples were used t-test, $p<0.05$, which was statistically significant.

Statistical analysis of changes in *EGFR* mRNA content in animal liver

Group	Number of examples	*M(P25~P75)*	*t*	*p*
Control	5	0.960(0.925,1.030)	-2.611	0.009
Anxiety	5	2.025(1.728,2.536)		

The normality test results of group C and group A were 0.960 and 0.971, respectively, satisfying normality, and the homogeneity test result was 0.025, which did not satisfy the homogeneity of variance, and two independent samples were used nonparametric Mann-Whitney U, $p<0.05$, which were statistically significant.

Statistical analysis of changes in EGFR protein content in animal liver

Group	Protein	Number of examples	$\bar{x}\pm s$ /M(P25~P75)	t/z	p
Control	p-EGFR	6	0.915±0.240	-2.986	0.014
Anxiety		6	1.245±0.128		
Control	EGFR	6	0.197±0.031	1.767	0.35
Anxiety		6	0.158±0.045		
Control	p-EGFR	6	4.775(3.575,5.624)	-2.882	0.004
Anxiety	/EGFR	6	7.754(6.248,10.471)		

The results of p-EGFR protein normality test in group C and group A were 0.609 and 0.848, respectively, which met the normality, and the homogeneity test result was 0.368, which satisfied the homogeneity of variance, and the difference was statistically significant by using two independent samples t-test, $p<0.05$. The results of EGFR protein normality test in group C and group A were 0.410 and 0.255, respectively, satisfying normality, and the homogeneity test result was 0.351, satisfying homogeneity of variance, and two independent samples were used t-test, $p<0.05$, which was statistically significant. The normality test results of p-EGFR/EGFR in group C and group A were 0.033 and 0.048, respectively, which did not meet the normality, and the homogeneity test result was 0.160, which satisfied the homogeneity of variance, and two independent samples were used nonparametric Mann-Whitney U, $p<0.05$, which were statistically significant.

Statistical analysis of the mean optical density of p-EGFR immunohistochemistry in animal liver

Group	Number of examples	$\bar{x}\pm s$	t	p
Control	8	200.036±6.878	5.820	0.000
Anxiety	8	219.931±6.793		

The normality test results of group C and group A were 0.084 and 0.320, respectively, satisfying normality, and the homogeneity test result was 0.658, satisfying the homogeneity of variance, and the $p<0.05$ was used by the t-test of two independent samples, which was statistically significant.

Statistical analysis of the mean fluorescence intensity of p-EGFR immunofluorescence in animal liver

Group	Number of examples	M(P25~P75)	z	p
Control	8	8.762(8.069,10.621)	-2.626	0.009
Anxiety	8	32.398(13.577,67.678)		

The normality test results of group C and group A were 0.000 and 0.041, respectively, which did not meet the normality, and the homogeneity test result was 0.010, which did not meet the homogeneity of variance, and two independent samples of nonparametric Mann-Whitney U, $p<0.05$ were used, and the difference was statistically significant.

Statistical analysis of changes in *PIK3R1* mRNA content in animal brains

Group	Number of examples	$\bar{x}\pm s$	t	p
Control	5	0.972±0.056	-4.582	0.002

Anxiety	5	1.333±0.167

The normality test results of group C and group A were 0.960 and 0.764, respectively, satisfying normality, and the homogeneity test result of variance was 0.054, satisfying the homogeneity of variance, and two independent samples were used t-test, $p<0.05$, which was statistically significant.

Statistical analysis of changes in PI3K protein content in animal liver

Group	Protein	Number of examples	$\bar{x}\pm s$/ $M(P25\sim P75)$	t/z	p
Control	p-PI3K	6	0.516(0.482,0.664)	-2.882	0.005
Anxiety		6	0.818(0.752,0.931)		
Control	PI3K	6	0.402±0.068	-0.884	0.398
Anxiety		6	0.451±0.117		
Control	p-PI3K	6	1.393±0.173	-3.555	0.005
Anxiety	/PI3K	6	1.918±0.328		

The normality test results of p-PI3K protein in group C and group A were 0.047 and 0.669, respectively, which did not meet the normality, and the homogeneity of variance test result was 0.919, which satisfied the homogeneity of variance, and two independent samples were used Mann-Whitney U, $p<0.05$, which were statistically significant. The results of PI3K protein normality test in group C and group A were 0.677 and 0.178, respectively, which met the normality, and the homogeneity test result was 0.162, which satisfied the homogeneity of variance, and the difference was statistically significant by using two independent samples t-test, $p<0.05$. The normality test results of p-PI3K/PI3K in group C and group A were 0.450 and 0.962, respectively, satisfying normality, and the homogeneity test result was 0.194, satisfying the homogeneity of variance, and the $p<0.05$ was used for the t-test of two independent samples, which was statistically significant.

Statistical analysis of changes in *AKT1* mRNA content in animal brains

Group	Number of examples	$\bar{x}\pm s$	t	p
Control	5	0.972±0.056	-3.229	0.012
Anxiety	5	1.251±0.185		

The normality test results of group C and group A were 0.960 and 0.591, respectively, satisfying normality, and the homogeneity test result was 0.064, satisfying the homogeneity of variance, and the two independent samples t-test, $p<0.05$, were statistically significant.

Statistical analysis of changes in AKT protein content in animal liver

Group	Protein	Number of examples	$\bar{x}\pm s$	t	p
Control	p-AKT	6	0.852±0.189	-2.008	0.072
Anxiety		6	1.043±0.135		
Control	AKT	6	1.464±0.254	1.047	0.320
Anxiety		6	1.340±0.136		
Control	p-AKT	6	0.588±0.130	-3.186	0.010
Anxiety	/AKT	6	0.778±0.064		

The normality test results of p-AKT protein in group C and group A were 0.456 and 0.463, respectively,

satisfying normality, and the homogeneity test result was 0.291, satisfying the homogeneity of variance, and the difference was statistically significant by using two independent samples t-test, $p<0.05$. The results of AKT protein normality test in group C and group A were 0.725 and 0.559, respectively, satisfying normality, and the homogeneity test result was 0.137, satisfying homogeneity of variance, and the difference was statistically significant by using two independent samples t-test, $p<0.05$. The results of p-AKT/AKT normality test in group C and group A were 0.310 and 0.791, respectively, satisfying normality, and the homogeneity test result was 0.156, satisfying homogeneity of variance, and two independent samples were used t-test, $p<0.05$, which was statistically significant.

Statistical analysis of changes in *NFKBIA* mRNA content in animal liver

Group	Number of examples	*M(P25~P75)*	*z*	*p*
Control	5	0.960(0.925,1.025)	-2.611	0.009
Anxiety	5	1.492(1.431,2.380)		

The normality test results of group C and group A were 0.960 and 0.066, respectively, satisfying normality, and the homogeneity test result was 0.034, which did not satisfy the homogeneity of variance, and two independent samples were used nonparametric Mann-Whitney U, $p<0.05$, which were statistically significant.

Statistical analysis of changes in IKBα protein content in animal liver

Group	Protein	Number of examples	$\bar{x}\pm s$	*t*	*p*
Control	p-IKBα	6	0.461±0.592	-1.193	0.260
Anxiety		6	0.506±0.071		
Control	IKBα	6	1.154±0.127	2.337	0.042
Anxiety		6	0.887±0.249		
Control	p-IKBα	6	0.401±0.044	-3.548	0.005
Anxiety	/ IKBα	6	0.593±0.125		

The results of p-IKBα protein normality test in group C and group A were 0.089 and 0.661, respectively, which met the normality, and the homogeneity of variance test result was 0.577, satisfying the homogeneity of variance, and the difference was statistically significant by using two independent samples t-test, $p<0.05$. The results of IKBα protein normality test in group C and group A were 0.339 and 0.194, respectively, which met the normality, and the homogeneity test result was 0.450, which satisfied the homogeneity of variance, and the difference was statistically significant by using two independent samples t-test, $p<0.05$. The normality test results of p-IKBα/IKBα in group C and group A were 0.061 and 0.695, respectively, satisfying normality, and the homogeneity test result was 0.086, satisfying the homogeneity of variance, and the $p<0.05$ was used to test two independent samples t, which was statistically significant.

Statistical analysis of changes in *NFκB1* mRNA content in animal liver

Group	Number of examples	$\bar{x}\pm s$	*t*	*p*
Control	5	0.972±0.056	-3.078	0.015
Anxiety	5	1.141±0.109		

The normality test results of group C and group A were 0.960 and 0.413, respectively, satisfying normality, and the homogeneity test result was 0.070, satisfying the homogeneity of variance, and the $p<0.05$ was used for the t-test of two independent samples, which was statistically significant.

Statistical analysis of changes in p50 protein content in animal liver

Group	Protein	Number of examples	$\bar{x}\pm s$/ *M(P25~P75)*	*t/z*	*p*
Control	p-p50	6	0.563±0.082	-3.093	0.010
Anxiety		6	0.759±0.131		
Control	p50	6	0.341±0.031	1.549	0.152
Anxiety		6	0.305±0.047		
Control	p-p50	6	1.710(1.343,1.895)	-2.562	0.010
Anxiety	/p50	6	2.223(2.013,3.300)		

The results of p-p50 protein normality test in group C and group A were 0.920 and 0.446, respectively, satisfying normality, and the homogeneity test result of variance was 0.257, satisfying the homogeneity of variance, and two independent samples were used t-test, $p<0.05$, which was statistically significant. The results of p50 protein normality test in group C and group A were 0.137 and 0.768, respectively, satisfying normality, and the homogeneity test result was 0.319, satisfying the homogeneity of variance, and two independent samples were used for t-test, $p<0.05$, which was statistically significant. The normality test results of p-p50/p50 in group C and group A were 0.806 and 0.115, respectively, satisfying normality, and the homogeneity test result was 0.050, which did not meet the homogeneity of variance, and two independent samples were used nonparametric Mann-Whitney U, $p<0.05$, which were statistically significant.

Statistical analysis of the average immunohistochemical optical density of p-p50 in animal liver

Group	Number of examples	*M(P25~P75)*	*z*	*p*
Control	8	9.678(2.696,12.668)	-2.338	0.021
Anxiety	8	16.455(11.831,46.304)		

The normality test results of group C and group A were 0.807 and 0.058, respectively, satisfying normality, and the homogeneity test result was 0.009, which did not satisfy the homogeneity of variance, and two independent samples were used nonparametric Mann-Whitney U, $p<0.05$, which were statistically significant.

Statistical analysis of the mean fluorescence intensity of p-p50 immunofluorescence histochemistry in animal liver

Group	Number of examples	$\bar{x}\pm s$	*t*	*p*
Control	8	3.633±1.348	-8.159	0.021
Anxiety	8	12.706±2.842		

The normality test results of group C and group A were 0.687 and 0.313, respectively, satisfying normality, and the homogeneity test result was 0.228, satisfying the homogeneity of variance, and the $p<0.05$ was used

to test two independent samples, which were statistically significant.

Statistical analysis of changes in *RELA* mRNA content in animal brains

Group	Number of examples	$\bar{x}\pm s$	*t*	*p*
Control	5	0.972±0.056	-2.172	0.041
Anxiety	5	1.451±0.437		

The normality test results of group C and group A were 0.960 and 0.126, respectively, satisfying normality, and the homogeneity of variance test results were 0.089, satisfying the homogeneity of variance, and the $p<0.05$ was used for the t-test of two independent samples, which was statistically significant.

Statistical analysis of changes in p65 protein content in animal liver

Group	Protein	Number of examples	$\bar{x}\pm s/M(P25\sim P75)$	*t/z*	*p*
Control	p-p65	6	0.428(0.367,0.604)	-2.562	0.010
Anxiety		6	0.909(0.834,0.947)		
Control	P65	6	0.603±0.082	0.492	0.633
Anxiety		6	0.581±0.073		
Control	p-p65	6	0.668(0.553,1.184)	-2.082	0.037
Anxiety	/p65	6	1.577(1.330,1.767)		

The results of p-p65 protein normality test in group C and group A were 0.029 and 0.131, respectively, which did not meet the normality, and the homogeneity test result was 0.254, which satisfied the homogeneity of variance, and two independent samples were used nonparametric Mann-Whitney U, $p<0.05$, and the difference was statistically significant. The results of p65 protein normality test in group C and group A were 0.097 and 0.819, respectively, satisfying normality, and the homogeneity test result of variance was 0.745, satisfying the homogeneity of variance, and the difference was statistically significant by using two independent samples t-test, $p<0.05$. The normality test results of p-p65/p65 in group C and group A were 0.016 and 0.936, respectively, which did not meet the normality, and the homogeneity of variance test result was 0.404, which satisfied the homogeneity of variance, and two independent samples were used Mann-Whitney U, $p<0.05$, which were statistically significant.

Statistical analysis of the immunohistochemical
mean optical density of p-p65 protein in animal liver

Group	Number of examples	$M(P25\sim P75)$	*z*	*p*
Control	8	7.460(4.492,8.547)	-2.195	0.001
Anxiety	8	13.026(11.586,13.641)		

The normality test results of group C and group A were 0.782 and 0.001, respectively, which did not meet the normality, and the homogeneity of variance test result was 0.769, which satisfied the homogeneity of variance, and two independent samples were used nonparametric Mann-Whitney U, $p<0.05$, and the difference was statistically significant.

Statistical analysis of the mean fluorescence intensity
of p-p65 immunofluorescence histochemistry in animal liver

Group	Number of examples	*M(P25~P75)*	*z*	*p*
Control	8	0.480(0.234,0.566)	-3.363	0.001
Anxiety	8	32.388(13.577,67.678)		

The normality test results of group C and group A were 0.556 and 0.041, respectively, which did not meet the normality, and the homogeneity of variance test result was 0.004, which did not meet the homogeneity of variance, and two independent samples were used nonparametric Mann-Whitney U, $p<0.05$, and the difference was statistically significant.

Statistical analysis of changes in inflammatory factor protein content in animal liver

Group	Protein	Number of examples	$\bar{x}\pm s$/*M(P25~P75)*	*t/z*	*p*
Control	TNF	6	0.573±0.164	-3.722	0.004
Anxiety		6	0.858±0.090		
Control	IL-1β	6	0.604(0.530,0.706)	-2.242	0.025
Anxiety		6	0.763(0.742,0.933)		
Control	IL-6	6	0.735±0.145	-5.481	0.000
Anxiety		6	1.101±0.076		

The normality test results of TNF protein in group C and group A were 0.545 and 0.440, respectively, which met the normality, and the homogeneity test result was 0.216, which satisfied the homogeneity of variance, and the difference was statistically significant by using two independent samples t-test, $p<0.05$. The normality test results of IL-1β protein in group C and group A were 0.915 and 0.033, respectively, which did not meet the normality, and the homogeneity test result was 0.891, which satisfied the homogeneity of variance, and two independent samples were used nonparametric Mann-Whitney U, $p<0.05$, which were statistically significant. The results of IL-6 protein normality test in group C and group A were 0.406 and 0.438, respectively, satisfying normality, and the homogeneity test result was 0.347, satisfying the homogeneity of variance, and the difference was statistically significant by using two independent samples t-test, $p<0.05$.

Statistical analysis of changes in the content of inflammatory factors in animal serum

Group	Protein	Number of examples	$\bar{x}\pm s$	*t*	*p*
Control	TNF	6	44.231±1.044	-6.796	0.000
Anxiety		6	53.573±2.890		
Control	IL-1β	5	3.962±0.593	-2.672	0.028
Anxiety		5	5.543±1.182		
Control	IL-6	6	16.471±2.076	3.650	0.004
Anxiety		6	23.689±3.527		

The normality test results of TNF protein in group C and group A were 0.079 and 0.457, respectively, which met the normality, and the homogeneity test result was 0.117, satisfying the homogeneity of variance, and the $p<0.05$ of two independent samples were used to test the t, which was statistically significant. The results of IL-1β protein normality test in group C and group A were 0.122 and 0.666, respectively, satisfying normality, and the homogeneity test result was 0.175, satisfying the homogeneity of variance, and the difference was statistically significant by using two independent samples t-test, $p<0.05$. The results of IL-6

protein normality test in group C and group A were 0.224 and 0.234, respectively, satisfying normality, and the homogeneity test result was 0.337, satisfying homogeneity of variance, and two independent samples were used t-test, $p<0.05$, which was statistically significant.
